# A Pediatric Interprofessional Cardiac Intensive Care Unit Intervention: CICU Teams and Loved Ones Communicating (CICU TALC) is Feasible, Acceptable, and Improves Clinician Communication Behaviors in Family Meetings

**DOI:** 10.1007/s00246-024-03497-7

**Published:** 2024-05-03

**Authors:** Jennifer Walter, Douglas L. Hill, Arzu Cetin, Aaron DeWitt, Katie Kellom, William Quarshie, Heather Griffis, Justine Shults, Robert Arnold, Jennifer Tjia, Karen Puopolo, Martha A. Q. Curley, Chris Feudtner

**Affiliations:** 1 Clinical Futures, Children’s Hospital of Philadelphia, Philadelphia, PA, USA; 2 Justin Ingerman Center for Palliative Care, Children’s Hospital of Philadelphia, Philadelphia, PA, USA; 3 Cardiac Center, Children’s Hospital of Philadelphia, Philadelphia, PA, USA; 4 Policy Lab, Children’s Hospital of Philadelphia, Philadelphia, PA, USA; 5 Qualitative Research Core, Children’s Hospital of Philadelphia, Philadelphia, PA, USA; 6 Data Science and Biostatistics Unit, Department of Biomedical and Health Informatics, Children’s Hospital of Philadelphia, Philadelphia, PA, USA; 7 Department of Biostatistics, Epidemiology, and Informatics, Perelman School of Medicine, University of Pennsylvania, Philadelphia, PA, USA; 8 University of Pittsburgh Medical Center, Pittsburgh, PA, USA; 9 Department of Population and Quantitative Health Sciences, UMass Chan Medical School, Worcester, MA, USA; 10 Department of Family and Community Health, University of Pennsylvania School of Nursing, Philadelphia, PA, USA; 11 Children’s Hospital of Philadelphia Research Institute, Philadelphia, PA, USA

**Keywords:** Pediatric cardiac intensive care unit, Interprofessional teamwork, Family meetings, Team-family communication

## Abstract

Parents of children in the pediatric cardiac intensive care unit (CICU) are often unprepared for family meetings (FM). Clinicians often do not follow best practices for communicating with families, adding to distress. An interprofessional team intervention for FM is feasible, acceptable, and positively impacts family preparation and conduct of FM in the CICU. We implemented a family- and team-support intervention for conducting FM and conducted a pretest–posttest study with parents of patients selected for a FM and clinicians. We measured feasibility, fidelity to intervention protocol, and parent acceptability via questionnaire and semi-structured interviews. Clinician behavior in meetings was assessed through semantic content analyses of meeting transcripts tracking elicitation of parental concerns, questions asked of parents, and responses to parental empathic opportunities. Logistic and ordinal logistic regression assessed intervention impact on clinician communication behaviors in meetings comparing pre- and post-intervention data. Sixty parents (95% of approached) were enrolled, with collection of 97% FM and 98% questionnaire data. We accomplished > 85% fidelity to intervention protocol. Most parents (80%) said the preparation worksheet had the right amount of information and felt positive about families receiving this worksheet. Clinicians were more likely to elicit parental concerns (adjusted odds ratio = 3.42; 95%CI [1.13, 11.0]) in post-intervention FM. There were no significant differences in remaining measures. Implementing an interprofessional team intervention to improve family preparation and conduct of FM is locally feasible, acceptable, and changes clinician behaviors. Future research should assess broader impact of training on clinicians, patients, and families.

## Introduction

Parents of patients in the pediatric cardiac intensive care unit (CICU) experience severe distress during their child’s hospitalization [1, 2], often made worse by suboptimal communication with their child’s clinicians [3–5]. Parental understanding of their child’s heart disease is often rated lower by their clinicians than parental self-perception [6] and parents of children who die have a delayed appreciation of their child’s prognosis [7, 8]. When parents receive conflicting information from team members, this can also diminish the quality of communication and negatively impact parental decision making [9]. Research on how to optimize communication with families has been prioritized by several groups of experts [10, 11] and professional organizations recommend family meetings (FM) for better coordination of communication with families [11, 12]. FM are when families meet with a subset of their child’s clinicians to receive both information on their child’s condition [13] and emotional support from clinicians [14, 15], and provide an opportunity for involvement in the decision making process [14–17].

Despite recommendations for how to conduct meetings [18], most evaluations of FM demonstrate significant missed opportunities. Opportunities to elicit parental concerns and questions are often missed, despite research that demonstrates higher parental satisfaction when they participate in more meaningful ways [19]. Parents request support in how to communicate with their child’s clinicians and report apprehension when told the team recommends a family meeting, however, there are no evidence-based approaches for how to support parental preparation for a family meeting [20].

To optimize the preparation of interprofessional teams and parents for FM, we utilized an experience-based codesign with pediatric CICU clinicians and families to develop the intervention “CICU Teams and Loved ones Communicating” (CICU TALC) [21]. CICU TALC sought to increase clinicians’ skills when communicating with families and colleagues, institute processes that prioritized parental concerns and questions, clarify clinicians’ roles in the family meeting, and to help families know what to expect from the family meeting. The objective of this Medical Research Council Phase II pilot study [22] was to evaluate the feasibility of enrolling families and collecting family data, fidelity to the intervention protocol after training clinicians in CICU TALC, parents’ perception of acceptability of the intervention, and to determine if there was any impact on clinician communication behaviors of engaging parental involvement in FM.

## Methods

### Study Design and Setting

This pre- and post-test pilot intervention study was conducted at the Children’s Hospital of Philadelphia (CHOP), a large urban pediatric hospital with 32 dedicated cardiac intensive care unit (CICU) beds. Pre-intervention data was collected from December 2018 to March 2020 and post-intervention data was collected from July 2021 to September 2022. For context, in the CHOP CICU, it is typical practice for rounds to occur outside the patient’s room or at their bedspace in open bays with family caregivers invited to participate if they would like. There is no other typical interval of a planned FM independent of ones initiated by the clinical team due to a clinical change or when requested by the family. The Children’s Hospital of Philadelphia Institutional Review Board approved this study, and the study is registered on ClinicalTrials.gov (NCT03749330).

### Population

Three populations participated in this study: CICU patients, parents of patients, and CICU clinicians who participated in a family meeting for an enrolled patient. Inclusion criteria for patients were that they are < 18 years old, had been admitted to the CICU for at least 14 days, and were selected for a family meeting discussion. The rationale for the CICU length of stay criterion was that patients who had been admitted to the CICU for 14 days or longer were perceived by the CICU clinicians as having an extended stay, which warranted review and discussion with families. Parents were eligible if their child was chosen for a family meeting, they were a legal decision maker, ≥ 18 years old, and were English-speaking. While permission was obtained from any parent who participated in a family meeting because it was recorded, only 1 parent per child was consented and enrolled to complete a post-intervention survey. CICU Clinicians were included based on participation in a family meeting with an enrolled patient.

### Intervention

**Fig. 1:**
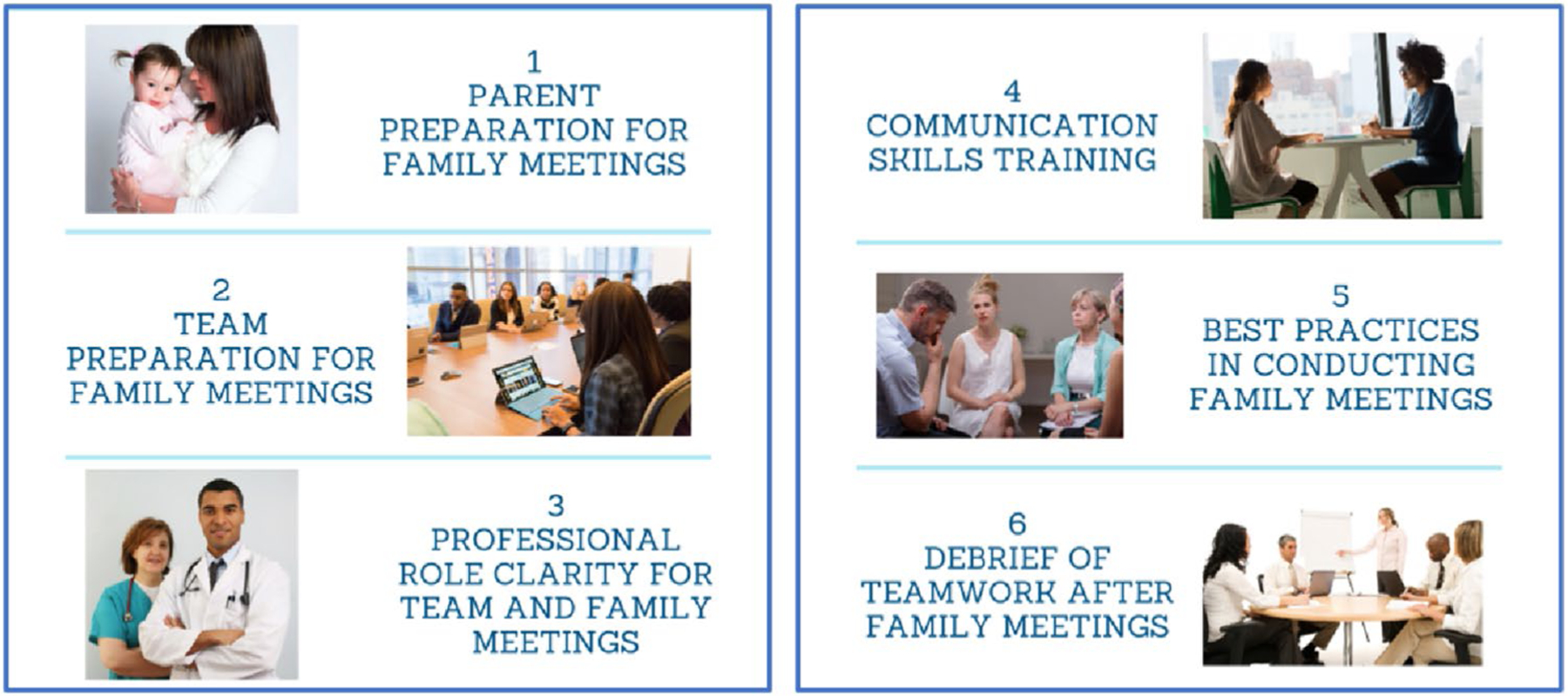
CICU TALC study components

An experience-based codesign was used with CICU clinicians and parents of children hospitalized in the CICU to develop the CICU Teams and Loved Ones Communicating (CICU TALC) intervention, including both clinician and parent facing elements and was described in detail elsewhere [21]. The intervention included both the development of processes to improve how teams and families prepared for and conducted family meetings and the implementation of communication skills training for interprofessional team members (Fig. 1). Communication skills training for clinicians focused on communicating serious news to families using a Vital Talk type training [23] and interprofessional communication skills training to optimize teamwork in communication within the team and the family. New team processes to better support the preparation for and conduct of FM by interprofessional teams included assigning a nurse to facilitate a pre-family meeting huddle to discuss the goals of the FM and incorporate all clinicians’ perspectives in developing a care plan and how to optimally communicate it to families. Four roles were assigned to clinicians for the FM to improve role clarity. These roles included a facilitator, information provider, emotional support coordinator, and someone to document the discussion. Finally, clinicians were trained in how to debrief the family meeting within a 5 min time frame to reinforce skills and promote future collaboration with team members.

The parent-facing components of CICU TALC included a handout provided in advance of the meeting to help parents prepare for their family meeting. The preparation worksheet aimed to inform them about what a family meeting is; the reason for their family meeting (described by one or more of five general categories: update on a prolonged hospitalization, new information, addressing conflicting information identified by the family, discharge planning, or decision about a medical treatment plan); and the likely questions that would be discussed in the meeting (e.g., what do you already understand about your child’s condition; what does it mean to be a good parent to your child). The worksheet also encouraged parents to write down their concerns about their child’s care, questions, communication preferences, and their preferences of which clinicians should attend the meeting. The social worker collected these questions and preferences from the family prior to the family meeting to share with the rest of the team in preparation for the meeting. After the family meeting, families received a summary sheet filled out by a clinician during the meeting. The summary sheet recorded all attendants’ names and roles, agenda items with corresponding action items, and items to be discussed later with other team members.

### Data Collection

The research team tracked participant eligibility, enrollment, data collection, completion rates, and fidelity to intervention protocol. Parental questionnaires collecting information about acceptability of CICU TALC were administered via REDCap after family meeting participation. Each family meeting was audio recorded and transcribed. A subset of parents was randomly selected for participation in a semi-structured post-intervention acceptability interview which was conducted in person or over the phone, audio recorded, and transcribed.

### Outcomes

Primary trial outcomes were feasibility of enrolling families, fidelity to the protocol, and parental perception of acceptability of CICU TALC. The secondary outcome was the impact of CICU TALC on clinician communication behaviors in FM comparing pre- and post-intervention FM.

#### Feasibility

Feasibility outcomes were measured by intervention enrollment numbers, retention, and family meeting data collection. Retention was assessed by examining the proportion of dyads who completed all data elements. Data collection was the amount of family meeting data collected compared to the intended data elements.

#### Fidelity to Intervention Protocol

The research team assessed fidelity of enactment to intervention protocol [24, 25] at post-intervention by evaluating meeting transcripts/recordings. Fidelity criteria that were defined prior to the data collection were (a) providing parents with the Preparation Worksheet before the family meeting, (b) following up with families about their communication preferences and questions for the team prior to the family meeting, and (c) provision of a Family Meeting Summary Sheet to the family after the family meeting.

#### Acceptability

Parents’ perceptions of the acceptability of the CICU TALC parent-facing materials were assessed using an adapted version of a previously validated acceptability satisfaction questionnaire [26] and semi-structured interviews. Open-ended interview questions were designed and piloted to solicit perspectives about the acceptability and utility of CICU TALC materials and the need for additional adaptation or improvement.

#### Clinician Behaviors

We assessed clinician behaviors in the FM using a modified VitalTalk coding scheme named The Studying Communication in Oncologist-Patient Encounters (SCOPE) [27–29]. Behaviors evaluated included elicitation of parental concerns, questions asked of the parents, and responses to empathic opportunities by parents when they expressed a negative emotion. We also tracked proportion of words spoken by professional group to determine if there was a shift in who contributed verbally to the family meeting.

### Statistical Analysis

We used descriptive statistics to summarize feasibility, fidelity, and parental questionnaire responses for acceptability. We followed COREQ guidelines [30] for qualitative data. An integrated approach [31] was used to analyze the parent interview data for acceptability, with a priori intervention-related codes as well as grounded theory codes that emerged from the interview transcripts. Study team members iteratively developed the code book by independently reviewing and comparing 16 successive transcripts. Once a common understanding of the codebook was established, 2 research coordinators coded the remaining interviews and resolved any coding disagreements through comparison and discussion.

A mixed methods approach was used to analyze the clinician behaviors in the FM. First, the FM were coded using the SCOPE codebook by trained study team members with double coding performed on at least 20% of transcripts. Discrepancies were resolved through comparison and discussion. We then evaluated the data for a quantitative count of the number of instances of each of the relevant outcome behaviors. Clinician behaviors on a meeting level were analyzed using the Fisher’s exact test and Pearson’s Chi-squared test for categorical/dichotomous variables and the Wilcoxon rank sum test for continuous variables. Pre and post-parent data was independent with different cohorts of participants and thus we could not perform statistical analysis to account for paired parental data. Outcomes that reached the prespecified threshold of significance (*P* value < 0.05) were then evaluated with logistic and ordinal logistic regression to assess CICU TALC impact on clinician communication behaviors at the meeting level comparing pre- and post-intervention data and controlling for number of participants in the meeting since the more people in the meeting, the less percentage talking time any one individual is likely to have.

Qualitative analyses for both the parent acceptability interviews and the clinician behavior in FM were performed in NVivo 13 [32]. Quantitative analysis was performed using the R (4.2.2) statistical software [33].

## Results

### Population

#### Patients and Parents

A total of 60 patients, each with a parent (for a total of 60 parents) participated in CICU TALC, of which 30 were in the pre-intervention and 30 in post. Demographics of the parents who consented to further data collection was 68% mothers, 58% White, and 77% were non-Hispanic (Table 1, top). Eighty percent of patients were less than 6 months old, 60% White, and 30% had a syndromic diagnosis (Table 1, bottom). The family meeting was conducted on CICU day of admission 35 (SD 29.8) in the pre-intervention and day 40 (SD 40.7) of the post-intervention. Additional parent and patient characteristics are in Supplemental Table A.

#### Families Present in Family Meetings

Even though only one parent per patient was officially enrolled in the study, more family members were allowed to participate in the FM. In a total of 58 FM, 72% had 2 parents present and on average 1.9 (median 2; range 1–4) family members (including parents, grandparents, siblings, and other support people). There was no difference in parent presence when comparing pre- and post-intervention FM.

#### Clinicians Present in Family Meetings

On average (mean) there were 4 clinicians in the pre-intervention (median 3.5; range 1–7) and post-intervention (median 3; range 2–6) meetings. In the pre-intervention, 96% of all meetings had a nurse present, in the post-intervention only 73% (due to COVID-19 pandemic staffing challenges). Social workers were present in 93% of all meeting (pre and post). The chaplain only participated twice in pre-intervention FM and once in a post-intervention family meeting. Out of 72 clinicians that participated in FM, 17 Clinicians (23.6%) were in both pre- and post-intervention FM (9 attendings, 4 social workers, and 5 nurses). 13 (18.1%) clinicians (7 attendings, 4 social workers, and 2 nurses) were trained in CICU TALC and participated in both pre- and post-intervention FM. 76% of all family meetings were led by one of the 7 trained attendings (82% in pre- and 70% in post-intervention). The 4 social workers that were trained participated in 91% of all FM (93% in pre- and 90% in post-intervention). Overall, at least one clinician (physician, social worker, or nurse) who completed the full intervention was present in each FM. All attendings who led the family meeting, even if they had not completed the training, were briefed in the elements of the intervention relevant to the family meeting prior to participation. Further information on clinician demographics is in Supplemental Table B.

### Feasibility

**Fig. 2:**
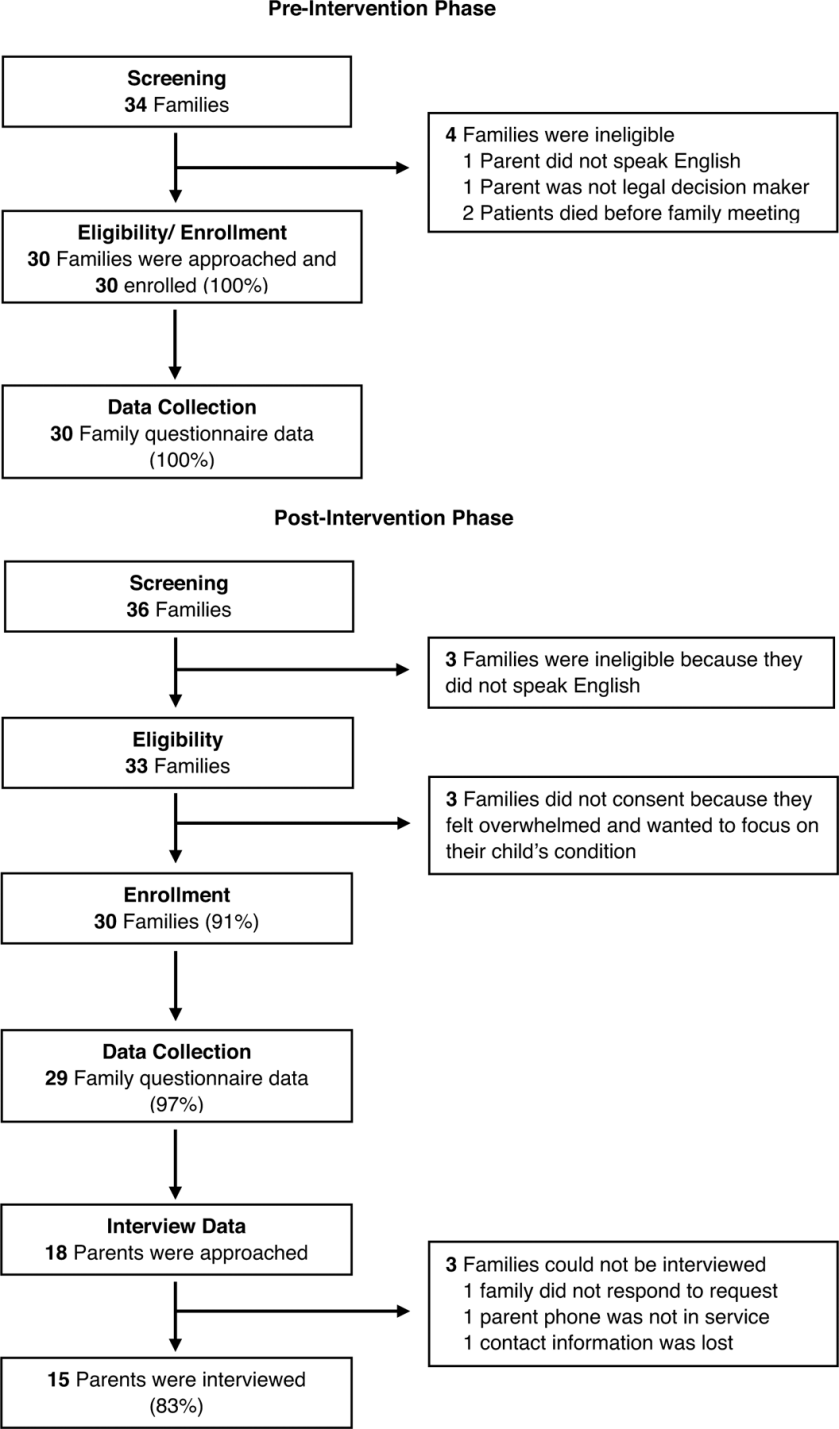
Participant screening, enrollment, and retention

A total of 63 patient-parent pairs were approached in the pre- and post-intervention (Fig. 2). We enrolled and collected questionnaire data from 100% of parents (*n* = 30) who were approached in the pre-intervention phase and from 93% (28/30) of the pre-intervention FM. We enrolled 91% (*n* = 30) of the 33 approached parents for the post-intervention FM. The three families that declined consent described feeling overwhelmed and wanted to focus on their child’s medical needs. Of these post-intervention parents, 97% (29) completed the questionnaire and we were able to collect 100% of family meeting data. In total, we were able to collect 98% of parent questionnaire data and 97% of family meeting data. On average the duration of FM in the pre-intervention phase was 49 min (SD 21) and 51 min (SD 19) in post-intervention.

### Fidelity of Intervention to Protocol

Intervention fidelity was monitored according to recommended practice [24, 25]. After evaluating the adherence to the intervention protocol for 8 successive post-intervention team and FM, the research team decided to provide augmented intervention delivery support to increase fidelity (see supplemental document C) with a study team member providing intervention element supports. Subsequently, enactment increased for all intervention measures (e.g., use of the summary sheet) above the pre-specified fidelity threshold of 90% after the delivery support was implemented (Table 2).

### Parent Perception of Intervention Acceptability

Demographic characteristics for parents who participated in interviews are in Supplemental Table D.

#### Preparation Worksheet

Post-intervention ratings of intervention acceptability were strong (Table 3). Most parents thought the length of the preparation worksheet was about right (88%) and had the right amount of information (80%). Most felt generally positive about parents receiving the preparation worksheet (80%). From the post-intervention interviews, qualitative themes and representative parent quotations are shown in Table 4. Themes surrounding the preparation worksheet included reduction of anxiety and help preparing and organizing thoughts. The only improvement parents suggested was that they would have liked to receive the preparation worksheet sooner to make better use of it.

#### Summary Sheet

Ninety six percentage of parents found the summary sheet helpful and were able to understand the summarized content. All parents said the summary sheet included the most important information discussed in the meeting (Table 3). In the acceptability interviews parents also expressed the pertinence and helpfulness of the summary sheet (Table 4). Some parents expressed that they would like the summary sheet to be more detailed, so they could use it as a clear reference point to evaluate their child’s progress.

#### Overall Family Meeting Experience

Overall, when parents first heard about the family meeting, they felt anxious but appreciated having a platform to voice their concerns (Table 4). After the meeting, parents felt re-assured that they had an opportunity to have their concerns heard and addressed.

### Clinician Behavior

Demographic characteristics for clinicians who participated in FM is described in Supplemental Table B. Bivariate results of clinician behaviors are demonstrated in Table 5. After adjusting for number of participants, clinicians were more likely to elicit parental concerns (OR = 3.42; 95%CI [1.13, 11.0]) in post-intervention FM compared to pre-intervention. There were no significant differences in the number of questions asked of parents or percent of words spoken by different professionals although social workers may have spoken more in post-intervention meetings (OR = 2.59 [95% CI 0.91, 7.73]) (Table 6).

## Discussion

Given the stress parents experience and the negative impact of communication breakdowns for families of children in the CICU, optimizing opportunities for interprofessional team communication with families is an important undertaking. These conversations can take a step back from the day-to-day management conversations to discuss the overall trajectory of care for the patient and clarify any misunderstandings that have occurred. Despite the potential benefits of family meetings, parents have anxiety about having a family meeting and can become overwhelmed in the meeting, reducing the ability to pose questions and absorb information [34]. Additionally, previous research has not demonstrated that family meetings impact parental perceptions of satisfaction with communication with the CICU team [35]. We hypothesized that families would benefit by having an opportunity to prepare for the meeting and by the team being aware of families’ questions in advance of the meeting. This CICU TALC intervention was designed with the goal of enhancing parental understanding, satisfaction with communication with the clinical team, and reducing parental anxiety [21].

This study demonstrated the feasibility and acceptability of implementing a complex team-based intervention for family meetings without extending their duration. Further, while not powered to assess efficacy, CICU TALC did correlate with greater elicitation of parental concerns, which is essential to ensuring family-centered care [34]. We were also able to collect relevant data from FM and parents and learned important lessons about how to ensure fidelity to the intervention protocol, all of which is central for future efficacy trials of CICU TALC.

Three aspects of the findings warrant discussion. First, our study demonstrated feasibility of implementing interprofessional team training with practicing clinicians even in high acuity settings at a single institution, which is consistent with other analyses of team trainings in the healthcare setting [36]. With high rates of parental enrollment, retention, and data collection we have demonstrated feasibility essential to drive future study of CICU TALC. Additionally, the implementation of complex behavioral interventions require careful monitoring of enactment (i.e., that the clinicians who were trained in CICU TALC performed the elements of the intervention) to ensure that the central elements of the protocol are implemented to be able to assess efficacy in future trials. [24] The need for an intervention delivery support to increase fidelity serves as a potential constraint for future implementation but may also demonstrate that future studies could be randomized at the patient level since teams will be unlikely to implement the intervention elements without ongoing support. Other studies providing support to adult surrogates of ICU patients have depended on the use of a person dedicated to ensuring study procedures are completed, similar to this pilot [37].

Second, we found substantial support and high rates of acceptability in both our quantitative and qualitative assessment of parental perception of CICU TALC. The intervention had been grounded in previous research which demonstrated that parents want to be included as valued members of their child’s care team and when that happens they are more likely to feel a sense of control, even in the face of prognostic uncertainty [20]. Additionally, CICU TALC aimed to provide guidance for parents on how to partner with their child’s providers and offered tangible supports to do so, an identified need of parents of children with congenital heart disease [38]. Even for parents who felt they were successful in understanding their child’s condition and advocating for them, the materials provided a useful double check in ensuring they were not missing anything and were doing all that they could. For families who were struggling to communicate effectively with teams, the materials provided an essential point of reflection to collect their thoughts and prepare for meetings and then with the summary sheet they could integrate what they learned to support future conversations.

Third, regarding potential efficacy of the intervention, while noting the small sample size of our pilot study we did find one statistically significant change in clinician behavior and several trends in the direction we would expect given CICU TALC’s goals. Clinicians significantly increased elicitation of parental concerns which was recommended as an important component of family-centered care in the ICU [12] and has been demonstrated to increase surrogate involvement in medical decision making [39]. There were also trends of more questions asked of parents to engage them further and more empathic responses to negative emotions expressed by families. Other research has demonstrated the limited number of empathic statements offered by clinicians in response to parents expressing their negative emotions [40] which hinders clinician ability to provide explicit support for families in times of high stress. The goal of increasing non-physician contributions in FM, which may also positively impact expressions of empathy and family engagement, was shown with a trend in social worker contributions in the meeting. This comes in contrast to data from multiple pediatric ICU settings where physicians dominated almost all of clinician communication [41, 42].

This pilot study had several limitations that bear discussion. First, CICU TALC was only implemented in one institution with a robust commitment to FM and adequate staffing to afford attendance by an interprofessional team. Second, the parent-facing materials were only available in English and the families who participated in the codesign were all English-speaking and did not consist of a representative population in the United States. Third, the pre- versus post-intervention study design was not able to account for all the changes in clinician and family experiences due to the COVID-19 pandemic, which encompassed none of the pre-intervention data collection and all the post-intervention data collection. In sum, future research should be done in other institutions with easily scalable elements like preparatory worksheets and summary sheets available in even poorer resourced hospitals; CICU TALC should be translated and culturally adapted for populations of families who may be at the highest risk for poor communication; and studies should employ a more rigorous study design to control for external events that have a broad impact on clinicians and families.

## Conclusion

An interprofessional team intervention in the pediatric CICU is feasible to implement and can be done so with high fidelity in a well-resourced setting. This intervention is also acceptable to English-speaking families, regardless of whether they perceive they are struggling to communicate effectively with their child’s healthcare team. Finally, CICU TALC may positively impact clinician behaviors in FM. These results warrant future efficacy testing of CICU TALC for impacts on parental understanding, satisfaction with communication, and stress.

## Supplementary Material

supplementary 2

Supplementary 1

supplementary 3

supplementary 4

## Figures and Tables

**Table 1:** Parent and patient characteristics

Parent Characteristics	Pre-Intervention *N* (%)	Post-Intervention *N* (%)	*P* value
Parent gender	*N* = 30	*N* = 29	
Female	18 (60%)	23 (77%)	0.11
Male	12 (40%)	6 (20%)	
Parent race	*N* = 30	*N* = 28	
White	19 (63%)	16 (57%)	0.90
Black or African American	7 (23%)	6 (21%)	
Other	4 (13%)	6 (21%)	
Parent Hispanic	*N* = 30	*N* = 27	
Hispanic	4 (13%)	7 (26%)	0.20
Not Hispanic	26 (87%)	20 (74%)	
Parent employment status	*N* = 30	*N* = 29	
Full time	24 (80%)	16 (53%)	**0.01** *
Part time	3 (10%)	1 (3%)	
Unemployed	3 (10%)	12 (40%)	
Parent relationship status	*N* = 30	*N* = 28	
Single	10 (33%)	11 (39%)	0.30
Married/partnered	20 (67%)	17 (61%)	
Total household income	*N* = 29	*N* = 28	
< $40,000	8 (28%)	5 (18%)	0.30
$40,001–$100,000	12 (41%)	11 (39%)	
More than $100,000	9 (31%)	12 (43%)	
Age at admission	*N* = 30	*N* = 29	
< 6 Months	26 (87%)	21 (72%)	0.23
6–12 Months	0 (0%)	2 (7%)	
More than 12 Months	4 (13%)	6 (21%)	
Patient gender	*N* = 30	*N* = 29	
Female	10 (33%)	11 (38%)	0.79
Male	20 (67%)	18 (62%)	
Patient race	*N* = 30	*N* = 28	
White	19 (63%)	16 (57%)	0.89
Black or African American	7 (23%)	6 (21%)	
Other	4 (13%)	6 (21%)	
Syndrome	*N* = 30	*N* = 29	
No	23 (77%)	18 (62%)	0.27
Yes	7 (23%)	11 (38%)	
Surgery this admission	*N* = 30	*N* = 28	
No	1 (3%)	1 (4%)	1.00
Yes	29 (97%)	27 (96%)	
STAT score	*N* = 26	*N* = 20	
Score 1–3	6 (23%)	8 (40%)	0.65
Score 4–5	20 (77%)	12 (60%)	
Surgical complication	*N* = 30	*N* = 29	
No	5 (17%)	1 (3%)	0.20
Yes	25 (83%)	28 (97%)	
Vasoactive-inotropic score at day 7 of admission	*N* = 0	*N* = 19	
< = 10	0 (0%)	17(89%)	NA
11 or more	0 (0%)	2 (11%)	
Major complication	*N* = 30	*N* = 29	
No	12 (40%)	12 (41%)	1.00
Yes	18 (60%)	17 (59%)	
Palliative care consultation	*N* = 30	*N* = 29	
No	21 (70%)	16 (55%)	0.29
Yes	9 (30%)	13 (45%)	

Chi-square tests or Fisher’s exact tests (when more than 20% of cells have expected frequencies < 5) results reported in Table 1

* *p* < 0.05

**Table 2:** Fidelity to intervention enactment

Measure	Intervention compliance score		
	All data, *N* = 30	Before augmented intervention delivery support, *N* = 8 (%)	After augmented intervention delivery support, *N* = 22 (%)
Social Worker gave a print or digital copy of the family meeting preparation worksheet (FMW) to the family in advance of the family meeting	27 (90.00%)	75.00	95.50
Social Worker followed up on the FMW and obtained questions the family would like answered in the meeting	26 (86.70%)	75.00	90.90
Clinician used the CICU family meeting summary sheet	27 (90.00%)	75.00	95.50

**Table 3:** Acceptability of family meeting worksheet among parents from questionnaires

	*N* (%)
Amount of information in the worksheet	*N* = 25
Less than I wanted	0 (0%)
About right	20 (80%)
More than I wanted	5 (20%)
Length of the worksheet	*N* = 25
Too short	0 (0%)
About right	22 (88%)
Too long	3 (12%)
Clarity of the worksheet	*N* = 25
Some things were unclear	1 (4%)
Most things were clear	10 (40%)
Everything was clear	14 (56%)
How you feel about families receiving the worksheet	*N* = 25
Negative	0 (0%)
Neutral	5 (20%)
Positive	20 (80%)
How worksheet was used (can select more than one)	*N* = 17
Information about family meetings	12 (17%)
Questions parent might be asked in meeting	4 (59%)
Prepared answers to questions parent might be asked	13 (76%)
Spoke to clinician prior to the family meeting about information from worksheet	3 (18%)
Clarity of CICU family meeting summary	*N* = 23
Some things were unclear	1 (4%)
Most things were clear	22 (96%)
Helpfulness of CICU family meeting summary	*N* = 23
Unhelpful	1 (4%)
Helpful	22 (96%)
CICU family meeting summary included most important information	*N* = 23
Yes	23 (100%)
No	0 (0%)
How parent used CICU family summary (can select more than one)	*N* = 23
Didn’t use	4 (17%)
Used when talking to other clinicians about what was discussed	2 (9%)
Used when talking to other family members about what was discussed	9 (39%)
Reviewed later to remind myself what was discussed	16 (70%)

**Table 4:** Parent Perception of Acceptability of Intervention from Interviews

Themes	Representative parent quotations
Preparation worksheet	
The preparation worksheet reduced parental anxiety about having a family meeting	“It gave me a very good idea of what they were gonna talk about because as I said, first we were a little confused, little scared. But then reading the [prep worksheet] and how [social worker] made it sound comfortable for us, so that was kinda good. Put us to ease.”
The preparation worksheet helped parents feel prepared	“[The prep worksheet] was helpful to help me come up with questions. Because at first, he’s like, ‘Well, do you have any questions?’ And my initial thought and response was, ‘No, not really.’ But once I read through the worksheet, questions did start popping up.”
Parents who did not think the worksheet was necessary still felt covered the right dimensions	“[The prep worksheet] was a good tool to use. Just, we didn’t need it. […] It asked the right questions. And was able to think about certain things, just those things we already thought about[…]”
Summary sheet	
Parents said the summary worksheet included the most important information discussed in the meeting	“Well, I mean as laymen to this whole medical realm, it is easy to get lost in the complexity and lost in the jargon. And I think having something short and simple to kind of bullet point the major issues that we’re dealing with as a family is helpful.”
Parents found the summary worksheet helpful as a reference to look back to when talking to family or providers	“It was definitely helpful so that I could…review what they said. Because there was a lot of information, so I’m sure I didn’t retain everything in the moment. But it was good so that I could like go back and my husband could see it, since he wasn’t there in person, but he had called in.”
Family experience in family meeting	
Parents appreciate having a platform to communicate their concerns	“I would encourage you to pursue continuing this type of meeting. I think this type of transparency and giving parents a platform to communicate their concerns and, fortunately for us, we’re pretty generally satisfied with the care. But it also gives parents the opportunity to address some things that may not be going well in other cases.”
Parents felt their concerns were listened to and addressed during the meeting	“And they wanted to answer whatever questions that I may have had. […] It was very reassuring. And they were very understanding and compassionate about my concerns.”
Suggested Improvements to Intervention	
Parents wanted to receive information (tools, materials, notice of the meeting) sooner	“I would say that it’s important to give the families enough time [to review the prep worksheet] because it is rather lengthy. And I think by necessity because there’s a lot of things we want to cover at these types of meetings. But this is definitely something that we needed to digest a week ahead of time. And slowly go through as a family to determine what our thoughts were for each of these questions or areas”
Wanted more information in the summary sheet	“Just so that’s something we’d be able to look back on and be like, oh okay. Well, two weeks ago this is where we were. And these were the bullet points on what exactly was keeping her in there, as of right now. And then you can sort of look at where you are now to compare it to where you were a couple of weeks ago.”

**Table 5:** Meeting level bivariate analyses of clinician behavior in family meetings

Characteristic	Pre-intervention *N* (%) *N* = 28	Post-intervention *N* (%) *N* = 30	*P* value
Total elicitation of parental concerns	8 (29%)	18 (60%)	0.016
Team elicits questions from family			
0	9 (32%)	3 (10%)	0.042
1–3	11 (39%)	21 (70%)	
4 or more	8 (29%)	6 (21%)	
	Median (IQR)	Median (IQR)	
Proportion of empathic responses to empathetic opportunity	0.50 (0.30, 0.72)	0.54 (0.35, 1.00)	0.3
Proportion of terminator responses to empathic opportunity	0.50 (0.28, 0.70)	0.46 (0.00, 0.65)	0.3
Total terminator responses	2.00 (1.00, 3.00)	1.00 (0.00, 2.00)	0.093
Proportion of words spoken by physician	0.64 (0.53, 0.78)	0.60 (0.52, 0.73)	0.40
Proportion words spoken by social worker	0.01 (0.00, 0.07)	0.04 (0.02, 0.07)	0.031
Proportion words spoken by parent	0.27 (0.13, 0.37)	0.29 (0.20, 0.34)	0.40
Proportion words spoken by nurse	0.04 (0.02, 0.09)	0.06 (0.02, 0.09)	0.7

**Table 6:** Pre-versus post-intervention family meeting process measures

Family meeting process measures	OR	95% CI	*P* value
1. Team elicits parental concerns (yes versus no)^a^			
Post-intervention	3.42	(1.13, 11.0)	0.029
Participants in attendance	0.85	(0.52, 1.36)	0.5
2. Team elicits questions from family^b^ (0 vs. 1–3, vs. 4 +)			
Post-intervention	1.89	(0.64, 5.79)	0.2
Participants in attendance	0.96	(0.62, 1.48)	0.9
3. Proportion of words spoken by social worker^b^ ([0, 0.01] vs. [0.01, 0.07] vs. > 0.07)			
Post-intervention	2.59	(0.91, 7.73)	0.076
Participants in attendance	0.82	(0.52, 1.26)	0.4

For models 1 and 2, *n* = 58, for model 3, *n* = 56

a Logistic regression modeling was used

b Ordinal logistic regression modeling was used

## Data Availability

No datasets were generated or analysed during the current study.
