# Supplementary material for: A Pediatric Interprofessional Cardiac Intensive Care Unit Intervention: CICU Teams and Loved Ones Communicating (CICU TALC) is Feasible, Acceptable, and Improves Clinician Communication Behaviors in Family Meetings
Source: Pediatr Cardiol. 2024 May 3;46(4):785–97. doi: 10.1007/s00246-024-03497-7 (PMC11531608; doi:10.1007/s00246-024-03497-7)
Supplement: Supplementary file 1 — Supplementary file1 (DOCX 30 kb) [file 246_2024_3497_MOESM1_ESM.docx]

**Supplemental Table D: Characteristics of Interviewed Parents**

|  | **N (%)** |
| --- | --- |
|  |  |
| **Parent gender** | **N=15** |
| Female | 11 (73%) |
| Male | 4 (27%) |
|  |  |
| **Parent age** | **N=15** |
| 18-29 | 6 (40%) |
| 30-39 | 6 (40%) |
| 40-50 | 3 (20%) |
|  |  |
| **Parent race** | **N=15** |
| White | 9 (60%) |
| Black or African American | 2 (13%) |
| Other | 4 (27%) |
|  |  |
| **Parent Hispanic** | **N=15** |
| Hispanic | 2 (13%) |
| Not Hispanic | 13 (87%) |
|  |  |
| **Parent employment status** | **N=15** |
| Full Time | 7 (47%) |
| Part Time | 1 (7%) |
| Unemployed | 7 (47%) |
|  |  |
| **Parent relationship status** | **N=15** |
| Single | 4 (27%) |
| Married/Partnered | 11 (73%) |
|  |  |
| **Total household income** | **N=15** |
| < $40,000 | 3 (20%) |
| $40,001 - $100,000 | 6 (40%) |
| More than $100,000 | 6 (40%) |
|  |  |
| **Parent education** | **N=15** |
| High School or less | 3 (20%) |
| Some College | 4 (27%) |
| College or Advanced Degree | 8 (53%) |
