# Supplementary material for: A Pediatric Interprofessional Cardiac Intensive Care Unit Intervention: CICU Teams and Loved Ones Communicating (CICU TALC) is Feasible, Acceptable, and Improves Clinician Communication Behaviors in Family Meetings
Source: Pediatr Cardiol. 2024 May 3;46(4):785–97. doi: 10.1007/s00246-024-03497-7 (PMC11531608; doi:10.1007/s00246-024-03497-7)
Supplement: Supplementary file 2 — Supplementary file2 (DOCX 14 kb) [file 246_2024_3497_MOESM2_ESM.docx]

**Supplemental Table B: Characteristics of Clinicians Who Participated in Family Meetings**

|  | **N (%)** |
| --- | --- |
|  |  |
| **Clinician gender** | **N=72** |
| Female | 55 (77%) |
| Male | 17 (24%) |
|  |  |
| **Clinician race** | **N=63** |
| White | 51 (81%) |
| Black or African American | 4 (6%) |
| Asian | 6 (10%) |
| Other | 2 (3%) |
|  |  |
| **Clinician Hispanic** | **N=58** |
| Hispanic | 3 (5%) |
| Not Hispanic | 13 (95%) |
|  |  |
| **Clinician Discipline** | **N=72** |
| Attending Intensivist | 13 (18%) |
| Cardiologist | 7 (10%) |
| Sub-specialist Fellow | 6 (8%) |
| Nurse Practitioner | 5 (7%) |
| Clinical Nurse Expert | 2 (3%) |
| Nurse Manager | 9 (13%) |
| Bedside Nurse | 16 (22%) |
| Social Worker | 5 (7%) |
| Other | 9 (13%) |
|  |  |
| **Clinician Years of Experience** | **N=54** |
| Less than 2 years | 6 (8%) |
| 2-5 years | 12 (17%) |
| 6-10 years | 12 (17%) |
| More than 10 years | 24 (33%) |
|  |  |
| **Participation in Family Meetings Per Week** | **N=51** |
| None | 10 (20%) |
| 1 | 33 (65%) |
| 2-4 | 8 (16%) |
