# Supplementary material for: A Pediatric Interprofessional Cardiac Intensive Care Unit Intervention: CICU Teams and Loved Ones Communicating (CICU TALC) is Feasible, Acceptable, and Improves Clinician Communication Behaviors in Family Meetings
Source: Pediatr Cardiol. 2024 May 3;46(4):785–97. doi: 10.1007/s00246-024-03497-7 (PMC11531608; doi:10.1007/s00246-024-03497-7)
Supplement: Supplementary file 3 — Supplementary file3 (DOCX 13 kb) [file 246_2024_3497_MOESM3_ESM.docx]

**Supplemental Document E: Intervention Delivery Augmentation by Study Coordinator**

1. Send Family Meeting Prep Worksheet to social workers for them to distribute.
2. Send physician leading medical discussion portion of the pre-meeting the PowerPoint template to facilitate their portion of the meeting and inform them who from nurse leadership or social work will be facilitating the family meeting preparation portion.
3. Attend nursing huddle to determine which nurse will be assigned to pre-meeting.
4. Send nurse leadership or social worker a template of their portion of the PowerPoint slides to facilitate the family meeting portion and the facilitation guide.
   - Intervention coordinator offers coaching to any members who haven’t facilitated before or have questions.
5. Explicitly reach out to bedside nurse to inform about meeting and provide virtual link to the meeting if nurse can’t leave the bedside.
6. Print the corresponding family meeting outline for the team meeting and distribute to team members.
7. Print the Summary worksheet and distribute to those team members who will be at the family meeting.
8. Collect the Summary Worksheet after family meeting to scan and copy.
   - Give a copy back to the family.
   - And upload a scanned version to electronic health record.
