# Supplementary material for: A Pediatric Interprofessional Cardiac Intensive Care Unit Intervention: CICU Teams and Loved Ones Communicating (CICU TALC) is Feasible, Acceptable, and Improves Clinician Communication Behaviors in Family Meetings
Source: Pediatr Cardiol. 2024 May 3;46(4):785–97. doi: 10.1007/s00246-024-03497-7 (PMC11531608; doi:10.1007/s00246-024-03497-7)
Supplement: Supplementary file 4 — Supplementary file4 (DOCX 14 kb) [file 246_2024_3497_MOESM4_ESM.docx]

**Supplemental Table A: Additional Parent and Patient Characteristics**

| **Parent Characteristics** | **Pre-Intervention**  **N (%)** | **Post-Intervention N (%)** | **P value** |
| --- | --- | --- | --- |
| **Parent age** | **N=27** | **N=29** |  |
| 18-26 | 5 (19%) | 6 (20%) | 0.5 |
| 26-40 | 19 (70%) | 17 (57%) |  |
| 41-50 | 3 (11%) | 6 (20%) |  |
|  |  |  |  |
| **Parent education** | **N=30** | **N=27** |  |
| High School or less | 7 (23%) | 5 (19%) | 0.7 |
| Some College | 7 (23%) | 8 (30%) |  |
| College or Advanced Degree | 16 (53%) | 14 (52%) |  |
|  |  |  |  |
| **Patient Characteristics** | **Pre-Intervention**  **N (%)** | **Post-Intervention N (%)** | **P value** |
| **Health insurance**** | **N=30** | **N=29** |  |
| Medicaid/other government | 18 (60%) | 12 (41%) | 0.20 |
| Private or employer plan | 15 (50%) | 19 (66%) | 0.30 |
|  |  |  |  |
| **Prior hospital admissions** | **N=30** | **N=29** |  |
| 0 | 6 (20%) | 10 (34%) | 0.48 |
| 1-2 | 15 (50%) | 12 (41%) |  |
| 3 or more | 9 (30%) | 7 (24%) |  |
|  |  |  |  |
| **Reason for Admission** | **N=30** | **N=29** |  |
| Medical Condition | 7 (23%) | 10 (34%) | 0.56 |
| Evaluation of Structural Heart Disease | 1 (3%) | 0 (0%) |  |
| Preop Cardiothoracic Surgery | 19 (63%) | 14 (48%) |  |
| Postop Cardiothoracic Surgery | 3 (10%) | 4 (14%) |  |
| Post Non-Cardiac Procedure | 0 (0%) | 1 (3%) |  |
|  |  |  |  |
| **Type of cardiac surgery** | **N=25** | **N=19** |  |
| Cardiopulmonary bypass surgery | 20 (80%) | 17 (89%) | 0.68 |
| Non-cardiopulmonary bypass surgery | 5 (20%) | 2 (11%) |  |
|  |  |  |  |
| **Ventilated at time of questionnaire** | **N=30** | **N=29** |  |
| No | 0 (0%) | 1 (3%) | 0.49 |
| Yes | 30 (100%) | 28 (97%) |  |
|  |  |  |  |
| **CICU length of stay current admission (median, IQR)** | 64.1 (36.5, 82.4) | 56.8 (41.8, 88.7) | 0.87 |
|  |  |  |  |
| **CICU Disposition** | **N=30** | **N=25** |  |
| Current hospital | 23 (77%) | 19 (76%) | 0.73 |
| Deceased | 4 (13%) | 5 (20%) |  |
| Home | 2 (7%) | 0 (0%) |  |
| Outside hospital | 1 (3%) | 1 (4%) |  |
|  |  |  |  |
| **Extracardiac abnormality** | **N=30** | **N=29** |  |
| No | 21 (70%) | 17 (59%) | 0.42 |
| Yes | 9 (30%) | 12 (41%) |  |
|  |  |  |  |
| **Chromosomal abnormality** | **N=30** | **N=29** |  |
| No | 21 (70%) | 15 (52%) | 0.19 |
| Yes | 9 (30%) | 14 (48%) |  |
|  |  |  |  |
| **Do not resuscitate order** | **N=30** | **N=29** |  |
| No | 30 (100%) | 26 (90%) | 0.11 |
| Yes | 0 (0%) | 3 (10%) |  |

*p<0.05. **Some patients had more than one form of health insurance.
